# The feasibility and acceptability of Project POWER: a mindfulness-infused, cognitive-behavioral group intervention to address mental and sexual health needs of young pregnant women in Liberia

**DOI:** 10.1186/s12884-023-05435-6

**Published:** 2023-03-20

**Authors:** Tamora A. Callands, Kandyce Hylick, Alethea Desrosiers, Shantesica M. Gilliam, Erica N. Taylor, Josalin J. Hunter, Nathan B. Hansen

**Affiliations:** 1 Department of Health Promotion and Behavior, College of Public Health, University of Georgia, Wright Hall, Room 321D, 100 Foster Road, Athens, GA 30606 USA; 2 Psychiatry and Human Behavior, The Warren Alpert Medical School, Brown University, Providence, RI USA; 3 Department of Environmental and Health Science, Spelman College, Atlanta, GA USA; 4 School of Social Work, College of Health and Human Sciences, University of North Carolina Wilmington, Wilmington, USA

**Keywords:** Mental health, Liberia, Pregnant women, Mindfulness, Sexual health, Intervention development

## Abstract

**Background:**

Following 14 years of civil war in Liberia, war exposure, gender-based violence, and extreme poverty have been identified as key challenges affecting the mental and sexual health of young pregnant women and the health of their unborn children. Despite ongoing efforts to rebuild the country’s healthcare infrastructure, empirical and culturally tailored interventions to address the consequences of war are severely limited. To address these concerns, we developed Project POWER (*Progressing Our Well-being, Emotions, and Relationships*), a mindfulness-infused, cognitive-behavioral intervention for young adult pregnant women. This study sought to 1) assess the feasibility and acceptability of POWER and 2) determine the preliminary efficacy of POWER for improving mental and sexual health outcomes among Liberian war-exposed young adult pregnant women.

**Methods:**

Eighty-seven women aged 18–25 were recruited from three catchment areas in Monrovia, Liberia to participate in a two-condition, pre-post design quasi-experimental pilot trial. Participants were allocated to the intervention (POWER) or the control condition (a health education program) based on where they resided relative to the catchment areas. Each condition completed a ten-session program delivered over 5-weeks. Feasibility and acceptability of POWER were examined using program logs (e.g., the number of participants screened and enrolled, facilitator satisfaction, etc.) and data from an end-of-program exit interview. The preliminary efficacy of POWER on mental and sexual health outcomes was assessed using repeated measures ANOVA with time and condition as factors.

**Results:**

Analyses provided preliminary support for the feasibility and acceptability of POWER. Participants attended an average of 8.99 sessions out of 10 and practiced material outside the sessions at least 2.77 times per week. Women in both conditions showed significant reductions in the level of prenatal distress (baseline, *M* = 16.84*,* 3-month assessment,* M* = 12.24), severity of post-traumatic stress disorder (PTSD) symptoms (baseline, *M* = 11.97*,* 3-month assessment, *M* = 9.79),), and the number of transactional sexual behaviors (baseline, *M* = 1.37*,* 3-month assessment, *M* = .94) over time. Participants who received POWER showed significant reductions in the frequency of depressive symptoms (baseline, *M* = 5.09*,* 3-month assessment, *M* = 2.63) over women in the control condition.

**Conclusions:**

Findings suggest that POWER may be a feasible and acceptable intervention to promote mental and sexual health for young adult pregnant women in Liberia. However, fully powered clinical trials are still needed to determine the efficacy and effectiveness of POWER before recommending its use on a larger scale in Liberia.

## Introduction

Exposure to traumatic events has been consistently linked to mental and sexual health problems in post-conflict settings [1–3]. Between 1989 and 2003, Liberia experienced a deadly civil war that resulted in over 150,000 deaths and war-related trauma exposure in 60% of the population [4]. Human rights violations (e.g., sexual violence and torture) were common during the war and have been associated with adverse mental and sexual health outcomes for many Liberians [5], including high rates of depression and post-traumatic stress [6]. Sexual and gender-based violence (SGBV) and unsafe sexual practices (e.g., transactional sex) during and post-conflict are also linked to increased rates of sexual and reproductive health problems [7, 8], including sexually transmitted infections (STIs), abortions, and unwanted pregnancies [5, 9]. Existing health services are still recovering from the war and are not equipped to address women's unmet mental and sexual healthcare service needs [3, 10–12].

For pregnant young women in Liberia and other low- and middle-income countries (LMICS), the need for integrated mental and sexual health services is high. Prenatal distress— stress, anxiety, or depression during pregnancy— is significantly higher in LMICs than in high-income countries [13]. Chronic distress throughout pregnancy places women at risk for neonatal and obstetric complications, such as premature birth, postpartum depression, and unplanned cesarean delivery [14]. In addition to negative impact on mothers, prenatal distress during pregnancy is associated with adverse perinatal outcomes among infants, such as low birth weight and preterm birth [15, 16]. Implementing mental and sexual health interventions for pregnant women in LMICs is essential for the health of women and their unborn children [17].

To address the need for integrated mental and sexual health services for young Liberian women, we developed and culturally adapted a mindfulness-infused cognitive-behavioral intervention called *Progressing Our Well-being, Emotions, and Relationships* (POWER) which sought to improve young pregnant women's mental and sexual health in Liberia (funded by NIH/FIC grant number 1K01TW009660). Strong evidence supports the effectiveness of mindfulness-based practices—paying attention in the present moment without judgment—in reducing anxiety, depression, and general psychological distress in pregnant women [18, 19]. The current study's objectives were: (a) to assess the feasibility and acceptability of POWER and (b) to evaluate the preliminary efficacy of POWER on mental and sexual health outcomes among war-exposed young adult pregnant women in Liberia.

## Methods

### Formative research

Project POWER evolved from a mixed-method study conducted between 2014 and 2017 in Monrovia, Liberia. The study assessed the mental and sexual health needs of young adult pregnant women in Liberia. The qualitative phase of the study consisted of five focus groups (approximately ten young adult pregnant or postpartum women in each group) and 17 key informant interviews. The interviews were conducted with mental health care providers, prenatal care/family planning service providers, and representatives from non-government organizations working in family planning, child welfare, or maternal and child health in greater Monrovia, Liberia. The quantitative phase of the study examined rates of intimate partner violence, sexual risk behavior, prenatal distress, transactional sex, depressive symptoms, PTSD symptoms, and life stressors among 200 pregnant women [1, 20, 21]. Results suggested that young pregnant women would benefit from developing skills that could reduce the impact of perinatal stress and depression and increase knowledge and skills to improve sexual health and relationships.

### Participant recruitment and procedures

Participants were recruited using purposive sampling in three catchment areas located in Montserrado county—the most populated county in Liberia—through a well-known local health clinic. Two catchments were allocated to the POWER condition and one to the control condition, as suggested by the clinic staff based on their knowledge of the communities and existing clinic programming at the local clinics. One nurse per clinic facilitated recruitment using existing clinic programming rosters to determine whether potential participants were involved in other programs within the clinic. If the women were not involved in other programs or were new to the clinic; and over 13 weeks gestation, their charts were flagged as potential participants. For those flagged during their prenatal care appointments, the nurse described the study and ascertained whether they would be interested in participating. If the potential participant expressed interest in the program, the nurse provided their contact information to a research team member, who then scheduled an in-person screening. In addition, the nurse asked potential participants if they knew other pregnant women (i.e., snowball sampling) who lived in their community that were due around the same time.

Two female research team members conducted the in-person screenings and administered the assessment battery for the study. Due to high illiteracy rates, the research team administered all questions using the computer-assisted personal interview (CAPI) by NOVA’s Questionnaire Development System. To reduce bias, the team members were not privy to the study inclusion criteria. The PI programmed the CAPI to render a decision of eligibility at the end of the screener. All data collection interviews took place at the main clinic, and the research assistants were unaware of the participant’s specific catchment areas. Before starting the interview, each participant was given a study ID number, which linked them to their catchment area and study condition. Research assistants were blinded to the study condition. While the assessments were taking place, we conducted a pre-pilot phase to inform and finalize intervention content with a matched sub-sample of young pregnant women recruited from the three catchment areas using the same recruitment strategy described above. Details of the pre-pilot phase are described in the study design section below.

Inclusion criteria included: (a) receiving prenatal or health services through the clinic; (b) 18–25 years old; (c) residing in Monrovia; and (d) between 13 and 24 weeks of gestational age. Exclusion criteria were: (a) pregnancy-related medical problems and (b) severe cognitive impairment (assessed via the mini-mental status exam). We used the gestation age of 13–24 weeks to ensure that women were past the first trimester, which comprises several risks and pregnancy-related decisions. This time frame also increased the likelihood that women completed the 3-month assessment before delivery.

Those interested in participating who met inclusion criteria and provided informed consent were enrolled in the study. Participants received an incentive equivalent to US $9 ($1 for travel, $3 for a meal, and $5 for completing assessments). Participation in the study was voluntary and confidential, and healthcare services were not affected by participation.

The study was approved by both US-based and Liberia-based Institution Review Boards: The University of Georgia’s Human Research Protection Program and The University of Liberia (UL)-Atlantic Center for Research and Evaluation (ACRE) Africa.

### Study Design

This quasi-experimental study used a two-condition (POWER and control), pre-post design to explore the feasibility, acceptability, and preliminary efficacy of POWER among young pregnant women. Both conditions completed quantitative assessments examining mental and sexual health at baseline (i.e., prior to the administration of the intervention) and then three months after the baseline assessment (approximately two months following completion of the intervention). Participants in the POWER condition also completed a semi-structured exit interview at the final session to assess experiences with the intervention (e.g., satisfaction with facilitators, how often they practiced skills outside of the program, etc.).

Three Liberian topical experts extensively reviewed all study materials for the POWER and control conditions before launching the pilot study. Experts were mental or sexual health services providers (i.e., social workers, nurses, midwives). To improve acceptability, topical experts suggested changes that fell into two areas: (1) local vernacular and language changes and (2) content changes. Several minor vernacular and language changes were made. For example, experts suggested using the term “itchy fish” to refer to a yeast infection and “man and women business” to refer to sexual intercourse in the sexual health module. The proposed content-related changes included a brief review of sexual anatomy in the sexual health session, including the female and male reproductive organs. In addition, experts suggested including a conflict resolution section as part of interpersonal relationships and communication skills session. All feedback from topical experts was incorporated into the intervention manual.

Facilitators for each condition (facilitators were teams of one nurse and one social worker) participated in a one-week training delivered by the program supervisor and the study PI to prepare for the delivery of the program material. Separate trainings were conducted for the POWER and control (health education) interventions. These in-person trainings included full coverage of all intervention sessions and a series of role-plays with each interactive activity.

After training, a two-week pre-pilot phase was conducted with a matched sub-sample of young pregnant women (lived-experience experts) for both conditions to gather feedback on the content, acceptability, and cultural relevance of intervention materials. The program supervisor provided oversight of the pre-pilot phase and helped refine intervention material based on participant feedback. This phase also enabled the facilitators (i.e., nurses and social workers) to practice facilitation skills, gain experience with the intervention material, and receive feedback on intervention delivery from participants and the supervisor.

The feedback from pre-pilot participants included making POWER activities more interactive and having the group meet twice a week for five weeks. For example, the emotional regulation session now consists of an activity where a group member draws a body, and each person comes to the board and draws a symptom of stress. No content suggestions were made for the control condition. Feedback from the pre-pilot phase was included in the final POWER and control condition intervention materials. Facilitators participated in weekly 90-min in-person supervision with the PI and program supervisor. Weekly supervision focused on [1] ensuring that facilitators were prepared to deliver upcoming program material, [2] discussions on how to ensure participants’ engagement with program material both in and out of sessions, and [3] providing instruction and using role-playing to address group facilitation difficulties.

#### POWER condition

POWER is a ten-session intervention delivered two times per week over five weeks. It incorporates core skills and concepts derived from evidence-based, mindfulness, and cognitive-behavioral therapy interventions to improve mental and sexual health outcomes [22–26]. These concepts include mindfulness-based activities such as mindfulness meditation, being present without judgment/acceptance, body scans, breathwork, and coping control. In addition, guided by WHO guidelines, the sexual health education components include knowledge and information regarding sexual health and relationships, skill development (e.g., how to use condoms, negotiating risk reduction strategies in a relationship), interpersonal support (e.g., ways to increase social support, increasing effective communication, and developing conflict resolution skills within an interpersonal relationship), and discovering and defining sexual values and attitudes [27, 28]. The primary goals of POWER are to [1] increase knowledge and awareness of the impact of mental health symptoms such as prenatal distress, depressive symptoms, and PTSD symptoms; [2] develop techniques to reduce or prevent mental health symptoms, including adaptive coping strategies and emotion regulation skills; [3] promote identification of personal sexual values and knowledge of sexual risk reduction strategies; and [4] increase knowledge and awareness of healthy interpersonal relationships (see Table 1 for more session details). The sessions were co-facilitated by one nurse and one social worker. Each session lasted approximately 90 min.

**Table 1:** Power Curriculum Outline and Connection to Guidelines

Session	Theme	Activities	Mindfulness/WHO guidelines
1	Introduction and trauma psycho education	• Establishing a safe group environment • Introduction and overview • Establishing group rules • Identifying personal goals • Finding a positive focus • Mindfulness meditation activity	• Presence w/o judgement • Acceptance • Mindfulness mediation
2	Emotion Regulation and Stress Management	• Opening the session • Introducing emotion regulation • Presenting coping strategies for stress management (Body scan activity) • Stress management activity (“Be good to yourself”) • Mindfulness meditation activity	• Body Scan • Coping control • Mindfulness mediation
3	Adaptive Coping	• Opening the session • Deep Breathing exercise • Introducing adaptive coping • “Let’s Talk It Out” Group Activity • Practicing adaptive coping techniques (muscle relaxation) • Mindfulness meditation activity	• Breathwork • Muscle relaxation • Coping control • Mindfulness mediation
4	Prenatal Distress/Mental Health	• Opening the session • Introducing maternal mental health and prenatal distress • Mindfulness meditation activity • Learning skills to reduce long-term stress and mental health problems	• Mindfulness meditation • Psychoeducation
5	Introduction to Sexual Health	• Opening the session • Introducing sexual health and sexually transmitted infections • Identifying ways to get tested • Sexual health-related activity • Mindfulness meditation activity	• Knowledge and information regarding sexual health • Risk reduction strategies • Mindfulness meditation
6	Sexual Values and Risk Reduction Knowledge	• Opening the session • Discussing the difference between power and pressure • dentifying sexual values • Learning about consent and healthy sexual decision making • Condom use activity • Mindfulness meditation activity	• Risk reduction strategies (skill development) • Condom use (skill development) • Sexual values and discovering attitudes about sex • Mindfulness meditation
7	Introduction to Healthy Relationships	• Opening the session • Identifying the characteristics of healthy and unhealthy relationships • Learning the benefits of healthy relationships • Relationship Activity • Mindfulness meditation activity	• Knowledge and information regarding healthy relationships • Mindfulness meditation
8	Interpersonal Relationships and Communication Skills	• Opening the session • Developing ways to increase social support • Developing and refining communication skills • Identifying communication styles • Learning strategies for conflict resolution (interpersonal relationships and listening) • Mindfulness meditation activity	• Increasing social support • Effective communication within interpersonal relationships • Conflict resolution • Mindfulness meditation
9	Relating to your Body and Baby After Birth	• Opening the session • Introduction to parenting • Learning strategies to effectively coping with new motherhood • Developing a viable support network with other parents • Learning techniques to combat common issues that arise with newborns (Breastfeeding, “Why is your baby crying?” activity) • Mindfulness meditation activity	• Mindfulness meditation • Coping control (new infant specific)
10	Building a Support Network and Celebration	• Opening the session • Discuss building a support network • Reviewing previous sessions • Celebrating the end of the program	• Interpersonal support • Mindfulness meditation

#### Control condition

There were no existing programs locally available that could serve as an attention-matched control condition, and a waitlist control introduced the potential confound of waitlist women giving birth during their intervention period following their wait period. Thus, building on knowledge gaps identified during the mixed methods study, our team developed an attention-matched general health education program for the control condition consisting of ten sessions delivered twice a week for five weeks. The general health curriculum provided information about the body and infectious disease control, hygiene, water and food safety, nutrition, substance use, mosquitos and malaria, hemorrhagic fevers, and infectious diseases. The sessions were co-facilitated by one nurse and one social worker. Each session lasted approximately 90 min.

## Measures

All study measures were culturally adapted using Beaton’s [29] guidelines for cross-cultural adaptation of self-reported measures. Due to high illiteracy rates, all measures were administered verbally to each participant using a computer-assisted program by a trained female research assistant. Our team collected demographics (i.e., age, level of education, relationship status, number of children), primary outcome measures (i.e., feasibility and acceptability), and secondary outcome measures (i.e., mental and sexual health symptoms) which participants completed at the baseline (pre-intervention) and 3-month assessments.

### Primary outcomes: feasibility and acceptability of power

#### Feasibility

We assessed the feasibility of POWER by examining the following: (1) feasibility of training and supervision (acceptability of training material and supervision and success in training facilitators to competence); (2) feasibility of intervention delivery within the clinic setting and within the project timeframe (logistical and organizational readiness for and success in intervention delivery and ability to cover intervention content and exercises within each session); (3) reach and enrollment (identification and screen of potentially eligible participants, enrollment of an adequate sample for trial); (4) attendance at intervention sessions and completion of study activity by participants (number of sessions attended, completing the baseline and 3-month assessments, and the exit interview); and (5) facilitators’ fidelity to intervention manual (coverage of session topics and skill development exercises, facilitator ratings of sessions). As an additional measure of fidelity, after each session, facilitators completed a session checklist that rated coverage of each session’s content and exercises using a Likert scale with items ranging from 1 (not covered) to 3 (fully covered), indicating adherence to the manual.

#### Acceptability

Program acceptability was operationalized as engagement with the content and satisfaction with the intervention process and content [30, 31]. Facilitators completed a checklist during each session that measured the engagement of each group member. The checklist used a Likert scale ranging from 1 (not engaged) to 3 (very engaged). In addition, the PI and topical experts developed a semi-structured exit interview to assess acceptability. It consisted of four quantitative and three qualitative questions. The four quantitative questions asked participants the following: (1) of the ten sessions, identify and rank the 3 most preferred sessions; (2) of the ten sessions, identify and rank the 3 least preferred sessions; and (3) how many times each week, did you practice coping skills (deep breathing, muscle relaxation, exercising, mindful meditations, body scans, listening to music, and talking it out) outside of the group (1 time per week to 7 or more times per week)?, and (4) rate your level of satisfaction with program facilitation using a 4-point Likert scale (“0” (not all satisfied) to “3 “(very satisfied)). The qualitative questions asked participants: (1) what did the facilitators do well throughout the program?; (2) what areas could the facilitators improve? (3) describe your thoughts and feelings about the program ending.

### Secondary outcome measures: mental and sexual health

#### Patient Health Questionnaire-9 (PHQ-9) [32]

The PHQ-9 assesses nine major depressive disorder symptoms defined by the DSM-IV. Participants were asked how often they experienced each symptom over the last two weeks. The responses were modified for this study based on suggestions from the cultural adaptation process. The original range was 0 (not at all) to 3 (nearly every day). The range was modified from 0 (not at all) to 2 (every day) to measure severity. Higher scores on the scale indicated that depressive symptoms were more severe. The Cronbach’s alpha was 0.72.

#### Prenatal Distress Questionnaire (PDQ)

The PDQ is an 18-item measure that assesses the extent to which respondents are concerned, troubled, or worried about issues related to their pregnancy [33]. Items are rated on a 3-point Likert scale, 0 (not at all) to 2 (every day). Item scores are summed to create a total score. Higher scores indicate higher levels of distress. The Cronbach’s alpha was 0.88.

*PTSD Symptoms (*Post Traumatic Stress Disorder Checklist-Civilian Version) (PCL) [34]*.* The PCL-C is a 17-item PTSD Checklist. Participants rated the severity of PTSD symptoms over the previous 30 days. The adapted response scale ranged from 0 (not at all) to 3 (all the time). The Cronbach’s alpha was 0.76.

#### Transactional Sex (TSC)

The TSC is a 9-item measure adapted from the Transactional Sex Scale [35]. The measure assessed three behaviors: 1) staying in a relationship longer than one wants; 2) starting a new relationship; 3) engaging in sex strictly for monetary/other goods. Participants were asked if they engaged in these behaviors for three reasons: (1) paying for items they could not buy themselves, (2) paying for food, children’s school fees, or taking care of the home, and (3) supporting children or family members who depend on them financially. The response choices were 1 (yes) or 0 (no). Scores are calculated by summing items. Participants were considered to have engaged in transactional sex if they indicated they received a score above zero. The Cronbach’s alpha was 0.82.

### Data analysis

First, descriptive statistics were computed to examine participants’ demographic characteristics and scores on the study variables at baseline (Table 2).

**Table 2:** Participant Characteristics at Baseline (*N* = 87)

	Overall	POWER	Control
	*n* (%)	`*n* (%)	*n* (%)
Age (M, SD)	20.72 (2.24)	20.33 (2.14)	21.14 (2.25)
Education			
No School	6 (7.9)	5 (11.1)	1 (2.4)
Primary (Grade 1–6) or less	21 (23.9)	8 (17.8)	13 (31.0)
Secondary (Grade 7–9)	30 (34.1)	20 (44.4)	10 (23.8)
High School (Grade 10–12 or greater)	30 (34.1)	12 (26.6)	18 (42.9)
Relationship status			
Not in a relationship	1 (1.1)	1 (2.2)	0 (0.00)
In a relationship	86 (97.7)	44 (97.8)	42 (100.0)
Any living children			
No	65 (73.9)	36 (80.0)	29 (69.0)
Yes	22 (24.9)	9 (20.0)	13 (31.0)
Number of times Pregnant			
1	57 (64.8)	33 (73.3)	24 (57.1)
2	18 (20.5)	6 (13.3)	12 (28.6)
3 or more	12 (13.6)	6 (13.3)	6 (14.3)
Outcomes (M, SD)			
Depressive Symptoms (PHQ) [0—18]	4.69 (4.01)	5.49 (4.53)	3.98 (3.38)
Prenatal Distress (PDQ) [0–48]	16.55 (11.40)	16.00 (10.83)	17.14 (12.10)
PSTD Symptoms (PCL) [0 -26]	12.09 (6.59)	13.07 (6.91)	11.02 (6.12)
Transaction Sex (TS) [0 -7]	1.23 (1.61)	.89 (1.17)	1.59 (1.92)

Next, the qualitative data from the exit interview was assessed by identifying themes and common responses. Each interview transcript was analyzed line-by-line, guided by grounded theory [36, 37]. A two-person team coded the transcripts collaboratively. A coding structure and codebook were created based on interview questions and participant responses [38, 39]. The codebook was developed and edited iteratively during data analysis until the primary investigator produced a final comprehensive codebook. Each interview was then coded or re-coded individually using the final codebook. The two-person coding team met regularly to discuss individual coding and code each interview collaboratively until 100% agreement was met. The PI then analyzed final coded text data across all interviews to yield salient themes using Nvivo qualitative analysis software.

Finally, to investigate whether the POWER intervention demonstrated preliminary efficacy in improving mental and sexual health outcomes, we performed a series of four two-way (time by condition) ANOVAs with time (baseline assessment and 3-month assessment) as the within-subjects factor and condition (intervention, control) as the between-subjects factor. Analyses were conducted using SPSS 25.0. Significance was established using two-tailed tests and an alpha level of 0.05. Only participants with complete baseline assessment and 3-month assessment were included in the analyses. Thus, twenty participants were dropped from analyses: one did not have complete pre-test data, and nineteen did not have complete post-test data.

## Results

### Participant characteristics

Eighty-seven women participated in the study (*n* = 45 in the POWER condition; *n* = 42 in the control condition). Table 2 displays sample demographic characteristics. Participants were 18–25 years old (*M* = 20.72, *SD* = 2.24). Almost eight percent of the sample had not attended school, approximately 24% had a primary school education, 34% had a secondary education, and 34% had a high school education or higher. The majority of the sample did not have living children (80% POWER (*n* = 36), 69% control (*n* = 29)). Only one participant indicated that she was not in a relationship.

### Primary outcomes: feasibility and acceptability of power

#### Feasibility

As noted above, facilitator teams consisting of a nurse and a social worker participated in a one-week training seminar conducted within a partnering organization. Feasibility of training was supported by the successful completion of training within this setting and facilitators displaying competence in delivery during role-plays of intervention sessions. Supervision was also successfully conducted with weekly supervision meetings with facilitators within the partnering organization. Qualitative feedback from the supervisor and facilitators indicated that supervision was helpful and acceptable. Additionally, the feasibility of intervention delivery within the clinic setting was demonstrated through ongoing organizational support of the program, good attendance by participants, and successful delivery of group sessions within the clinic.

We were very successful in reaching and enrolling the target population. Eighty-seven pregnant women between 18 and 25 were identified within partnering organizations and catchment communities and were invited to complete the screening interview for participation in the intervention study. All invited women consented to screening, met the study eligibility criteria, and were enrolled in the study (*n* = 87). The retention of participants in the group intervention and the overall study was good. The average number of sessions attended was 8.99 out of 10 for POWER participants and 8.02 out of 10 for control participants. Participants indicated that the clinic’s location in the local community greatly influenced their session attendance. The 3-month assessment completion rate was 79%. Finally, forty out of forty-five POWER participants completed the exit interview at the end of the intervention. An exit interview was not administered to the control group participants.

Facilitators successfully delivered the intervention and utilized supervision during the study. Delivering the intervention in the clinic setting, the length of intervention sessions (90 min), and the frequency of sessions (two sessions per week) were all logistically feasible and did not contribute to excessive burden on study participants, group facilitators, or the partner organization. Finally, supervisor reports and facilitator scores on session fidelity rating scales indicated excellent adherence to protocol. Fidelity rating scales yielded an average adherence score of 3.00 out of 3.00—meaning that the session content was delivered as prescribed in the manual 100% of the time for all sessions. 

#### Acceptability

The facilitator reports of group member engagement yielded an overall average score of 2.56 out of 3.00 across groups (i.e., group 1 (*M* = 2.47); group 2 (*M* = 2.64); group 3 (*M* = 2.55); group 4 (*M* = 2.56)) and sessions. Participants identified and ranked the sessions they liked most and least. The emotion regulation session was ranked highest (77.5%), followed by healthy relationships (47.5%) and communication skills (45%). 67.5% of participants selected not applicable’ when asked to identify and rank the top three least preferred sessions. The remaining participants identified and ranked sexual health, prenatal distress, and sexual values as the least preferred sessions. The adaptive coping skills most practiced outside of the group were: deep breathing (*M* = 2. 77 times per week), muscle relaxation (*M* = 3.05 times per week), mindfulness meditation (*M* = 3.15 times per week), talking to someone (*M* = 3.74 times per week), and listening to music (*M* = 3.77 times per week). Using a 4-point Likert scale ranging from 0 to 3, participants’ average rating for facilitation satisfaction was 2.51 (between satisfied and very satisfied).

Regarding the exit interview qualitative responses, when asked what the facilitators did well, *helpfulness* emerged as a primary theme. The helpfulness theme included reports that the facilitators took their time to ensure that the session information was explained well. Thus, participants found this helpful in terms of adaptive coping and motivation to practice skills. For instance, participants reported that (quotations are verbatim in Liberian English), “she (*the facilitator*) make me understand”; “she (*the facilitator*) always talks to me to help me manage my stress”; “she (*the facilitator*) is very encouraging people and explains the topics well”). A communication theme emerged from participant responses when asked what the facilitators could improve. The communication theme included reports that facilitators could have improved “how” the information was conveyed during facilitation. For instance, participants reported that (quotations are verbatim in Liberian English) facilitators “could speak and make her words clearer” and “could speak slower.”

The final question of the exit interview asked participants to describe their thoughts and feelings regarding the program’s end. The themes that emerged were “feeling good” and “feeling sad.” These themes indicate participants had mixed feelings about the program ending.

These themes suggest that many participants had mixed feelings about the program ending, with positive feelings about what they accomplished in the group and feelings of loss regarding the group coming to an end. For example, one participant said, “I feel good because it educated me along.” Other participants said, “I feel good and bad because the program empowered me by more knowledge…I feel bad because I won’t be seeing the program again”, and indicated a desire to continue learning (e.g., “I don’t want the program to end,” “I want to learn more”).

### Preliminary efficacy: mental and sexual health outcomes

The effect sizes for the secondary outcomes are provided in Table 3. These include Cohen’s D’s for time, condition, and time by condition estimates.

**Table 3:** Effect Sizes of Secondary Outcomes

	PHQ	PDQ	PCL	TS
	Partial η^2^	Partial η^2^	Partial η^2^	Partial η^2^
Time^a^	1.44***	.75**	.55*	.55*
Group	.58	.44	.00	.35
Time * Group	.80*	.06	.33	.59*

*Note*: ^a^ indicates that the data were collapsed across the baseline and 3-month assessments. *p < .05, **p < .01, ***p < .001

#### Depressive symptomology

Results of the ANOVA examining depression symptoms indicated there was no significant effect by condition [*F*(1,41) = 3.487, *p* = 0.069, *Cohen’s d* = 0.54]; however, there was a significant effect by time [*F*(1,41) = 21.179, *p* = 0.000, *Cohen’s d* = 1.44]. Specifically, symptoms of depression decreased from baseline (*M* = 5.09, *SD* = 4.14) to 3-month assessment (*M* = 2.63, *SD* = 2.70). In addition, there was a significant time by condition interaction [*F*(1,41) = 6.621, *p* = 0.014, *Cohen’s d* = 0.80]. A robust decrease in depressive symptoms was evidenced from baseline (*M* = 7.00, *SD* = 4.64) to 3-month assessment (*M* = 2.65, *SD* = 2.29) for those in the POWER condition. The decrease in depressive symptoms was much less robust for those in the control condition from baseline (*M* = 3.85, *SD* = 3.31) to 3-month assessment (*M* = 2.62, *SD* = 2.98).

#### Prenatal distress

Results of the ANOVA examining prenatal distress showed a significant effect of time [*F*(1,66) = 9.219, *p* = 0.003, Cohen’s *d* = 0.75]. Distress decreased from baseline (*M* = 16.84, *SD* = 11.17) to 3-month assessment (*M* = 12.24, *SD* = 9.49) for both conditions. There was no significant difference by condition [*F*(1,66) = 3.284, *p* = 0.75, *Cohen’s d* = 0.44], nor was there a significant time by condition difference [*F*(1,66) = 0.043, *p* = 0.836, Cohen’s *d* = 0.06].

#### PTSD Symptoms

Results of the ANOVA examining PTSD symptoms showed a significant effect of time [*F* (1,66) = 4.952, *p* = 0.29, *Cohen’s d* = 0.55]. PTSD symptoms decreased from baseline (*M* = 11.97, *SD* = 6.80) to 3-month assessment (*M* = 9.79, *SD* = 5.71) for all participants. There were no significant differences by condition [*F*(1,66) = 0.004, *p* = 0.952, *Cohen’s d* = 0.00], nor was there a significant time by condition interaction [*F*(1,66) = 1.862, *p* = 0.177, *Cohen’s d* = 0.33].

#### Transactional Sex

For transactional sex, results of the ANOVA indicated there was no significant effect by condition [*F*(1,66) = 2.039,* p* = 0.158, *Cohen’s d* = 0.35]; however, there was a significant effect by time [*F*(1,66) = 5.061, *p* = 0.028, *Cohen’s d* = 0.55] such that engagement in transactional sex decreased from baseline (*M* = 1.37, *SD* = 1.71) to 3-month assessment (*M* = 0.94, *SD* = 1.21) across all participants. In addition, there was a significant time by condition interaction condition [*F*(1,66) = 5.698, *p* = 0.020, *Cohen’s d* = 0.59]. Transactional sex decreased from baseline (*M* = 1.84, *SD* = 2.08) to 3-month assessment (*M* = 0.91, *SD* = 1.20) for the control condition [*F*(1,66) = 10.15, *p* < 0.001, *Cohen’s d* = 0.78]. Transactional sex remained stable for those in the POWER condition from baseline (*M* = 0.94, *SD* = 1.19) to 3-month assessment (*M* = 0.97, *SD* = 1.23) [*F*(1,66) = 0.01, *p* < 0.921, *Cohen’s d* = 0.00].

## Discussion

The current study explored the feasibility, acceptability, and preliminary efficacy of an innovative, mindfulness-infused, cognitive-behavioral intervention for young adult pregnant women in post-conflict Liberia. To our knowledge, POWER is the first mental and sexual health intervention with preliminary evidence for efficacy implemented in post-conflict Liberia. Study findings support the preliminary benefits of the intervention and the need for a larger, fully powered, randomized clinical trial to examine clinical efficacy, followed by effectiveness and implementation research that could enhance adoption and sustainment within Liberia's existing health service infrastructure. We successfully enrolled the target number of participants, and retention rates were acceptable. Participants reported high satisfaction with POWER sessions and reported practicing skills learned outside of the intervention sessions, including mindfulness meditation, muscle relaxation, deep breathing, and walking. Findings on intervention skills used most and least could inform further adaptations of POWER to best meet the needs of young pregnant Liberian women. For example, we could incorporate additional mindfulness-based activities, such as pregnancy-friendly yoga poses or focusing on bodily sensations.

POWER participants reported significant reductions in depression symptoms at the 3-month assessment. Findings consistently show that mindfulness-based interventions improve maternal depression, stress, and emotion regulation skills [40–42]. Though preliminary, findings may also have implications for children's health because maternal depression during pregnancy and postpartum has been strongly associated with poor child development outcomes [43, 44].

POWER and control participants reported significantly decreased prenatal distress and PTSD symptoms at the 3-month assessment. Though speculative, the focus on cultivating muscle relaxation and mindfulness skills may have helped participants increase their distress tolerance [19]. Given the adverse consequences of prenatal distress for maternal and child health, including adverse birth outcomes, lower likelihood of breastfeeding, and deficits in children's cognitive and behavioral function [13, 15], effective services targeting a reduction of distress during pregnancy are essential. Effect sizes suggest that a fully powered trial may have resulted in significant differences by condition. Findings also suggest that our health education curriculum may help reduce distress among young pregnant mothers and could potentially serve as an adjunct or alternative resource to the POWER intervention.

Participants reported significant reductions in the frequency of transactional sex at the 3-month assessment, but the time-by-condition interaction was not in the hypothesized direction. Transactional sex decreased for those in the control condition only. One possible explanation for this finding is that women in the POWER condition had low scores on transactional sex at baseline (mean of 0.89, indicating each participant had less than one indicator of transactional sex, on average), which may have produced a floor effect as scores had little room to improve. As a result, their scores may have been more likely to remain stable from baseline to the 3-month assessment. On the other hand, women in the control condition had higher scores (mean of 1.59, indicating that each participant had between one and two indicators of transaction sex, on average). While transactional sex scores between conditions did not differ statistically at baseline, there is a potentially meaningful difference between these scores. Regression to the mean is also a plausible explanation for observed outcomes. Another potential explanation for why transactional sex decreased for women in the control condition is that the control intervention provided information about general health, hygiene, and how to protect one’s body from harm. Women also received social support from other group members. Further adaptations and refinements to POWER may be necessary to target better reducing transactional sex, such as education about the health risks of transactional sex or strategies to secure income in healthier ways (e.g., economic empowerment).

### Limitations

Study results should be viewed in light of several limitations. The sample size was small and not powered to determine statistically significant treatment effects or generalize to other populations. The longer-term benefits of POWER were also not evaluated. Including a longer follow-up period in a future, fully-powered efficacy trial would provide more information on whether intervention benefits are sustained over time and whether there are benefits for early childhood development outcomes. Additionally, because participants were not randomly assigned to conditions, there is the potential for sampling and selection biases.

## Conclusion

The preliminary finding of POWER support the feasibility, acceptability, and mental and sexual health benefits of the intervention for young pregnant Liberian women. Further investigation of POWER on a larger scale is needed to determine whether the intervention is efficacious. With additional evidence for POWER’s effectiveness, the intervention could help increase access to evidence-based sexual and mental health services for young pregnant women in Liberia and other post-conflict settings, which has the potential to improve women’s health and the health of future generations [17].

## Data Availability

The data used during the current study are available by reasonable request from the corresponding author.
